# Gunshot Wound of the Vertebra

**Published:** 1906-09

**Authors:** H. A. Moseley

**Affiliations:** Dallas, Texas


For Texas Medical Journal.

### Gunshot Wound of the Vertebra.

BY H. A. MOSELEY, M. D., DALLAS, TEXAS.

DALLAS, TEXAS, August 13, 1906.

*Editor Texas Medical Journal, Austin, Texas:*

On the evening of April 27, 1906, about 11 o'clock, as Mr. Grover Record and a young lady were returning from the skating rink at the Fair Grounds they were attacked by a Mr. Walker, an admirer and lover of the young lady. Walker having secreted himself in the shadow of a neighbor's barn, shot the young lady and also Mr. Grover Record, using a 38-caliber pistol. The wound of the young lady was a simple flesh wound. In ten days she had recovered and was able to return to her school duties. Walker made a more successful shot, and put an end to himself.

In Grover Record's case, the ball entered midway between the crest of ilium and the rib, rather anteriorly, passing through to the spine, fracturing the lamina and a portion of the body of the sixth lumbar vertebra so that it pressed on the cord, causing complete paralysis of the body from the vertebra down, also greatly affecting the bowels and kidneys, as well as the power of locomotion. He was taken immediately to a sanitarium and kept there until the two attending surgeons were satisfied that the ball had entered the bowel and had passed out per rectum. This was ascertained by means of the X-ray apparatus [ ?]. They ordered patient sent to his place of abode.

Mr. Record having been raised near my old home in Tennessee, and having a great deal of confidence in my opinion, requested his friends to send for me. He being still under the care of his former physicians, I refused to call. However, that evening the boy, Record, was brought to my office for my opinion, which I gave after calling in Dr. J. B. Norris, whom I associated with me in the case.

We placed Record under the X-ray and soon located the ball imbedded in the sixth lumbar vertebra, impinging so deeply in the cord as to demand its immediate removal. This was done in the private sanitarium of Dr. J. H. Reuss, who assisted Dr. J. B. Norris and myself in the removal of the ball, which had been located by the skiagraph of Dr. Norris. Afterward several pieces of the lamina and other fragments of bone were removed to prevent injury to the cord in after-treatment. It was fully eight weeks before he was permitted to remain in an erect position.

It was certainly a very remarkable case, for within ten days there was perceptible sensation in the extremities, and a gradual return, so that by the 12th of August the young man was able to report to his employers for work. This shows another grand success attributed to the X-ray and antiseptic surgery.

I take pleasure in commending Dr. J. M. Reuss, father of Dr. J. H. Reuss, who is in his 84th year. He administerer the chloroform in a most successful manner, it being his 2000th case.

Yours truly,

H. A. Moseley.

[The ball extracted and the portions of the lamina can be seen at the office of Dr. H. A. Moseley.—Ed.]

---

The "Occult Eye" Wants "Light."—The following letter, very suggestive of Silas Wegg, is submitted as a rare specimen of that rarest of all humor—the unconscious and unintentional:

THE OCCULT EYE MAGAZINE CO.

J. C. Jones, M. D. Manager. Office 217 V Street N. W.

Washington, D. C., August 16th, 1906.

*"Personal."*

The Editor or Manager
Of
The Texas Medical
Journal.
Austin, Tex.

Dear Sir:—kindly send me a sample copy of the journal by return mail, likely to become a *subscriber*. Also kindly furnish me *some information,*

Do the Medical Board of Examiners interrupts a registered Physician & Surgeon in this state compelling them to re-register, or take the Board-Examination, Who have lawfully registered under the Old-Law. before the New-Law Existed or not, being already qualified Under the Old registration act. Is the Old-Law Retro-active, or have it been appealed and Amended, so as to effect any physician of the class just quoted. Is not this *board* is compell to issue their Certi*ficate* to said class *physicians* without Examinations, free of Charge, &Etc., Without adhering or blocking out any particular school of Practice. Do the Old-Law Certificate holds good, *Valid or not* or can this board have such Certificates revoked, from a Physician please give me full Light on these questions by return mail.

Explain fully any disadvantages experienced or would be Experienced by a practitioner who registered Under the Old-Law regardless of College of graduation, or System of Practice, Who now would enter this State to practice or begin his professional practice. some states *once registered always,* the New Law, never effects them, or Evade them or debarr them in his Medical practice or Law-practice, give me full details, & are the State Societies a benefit in such cases. and if taking too much of your time give a brief, Explanatory Idea, let me hear at once please.

from a fellow citizen
With Many thanks for your advanced favor
Sincerely yours,
J. C. Jones, M. D,
At present address No 217-V. St. N. W.
please also, favor me Washington, D. C.
and Send me a list of associations, & Cities societies
The State Medical & County Societies, of Texas and Fees
also requirements to enter.
of course if published in your Journal. so Much the better.
yrs
Jones, M. D.
please accept thanks
again.

Stamps Enclose

---

The Annual Yellow Fever Scare Is On.—It has about lost all its terrors, however. Small outbreak reported at New Iberia, La. Tabor is on deck and all is quiet along the Sabine.

